# An Interesting Case of Cerebral Tumour

**Published:** 1908-05-30

**Authors:** D. M. Macdonald

**Affiliations:** Dunkeld.


### THE GENERAL PRACTITIONER'S COLUMN.

[Contributions to this Column are invited, and if accepted will be paid for.]

### AN INTERESTING CASE OF CEREBRAL TUMOUR.

By D. M. MACDONALD, M.D., Dunkeld.

On February 23, 1907, W. H., aged 60, by occupation a gardener, was quite suddenly seized with severe twitchings of the muscles, first of one, the right, then of both sides of the face. These lasted for about an hour at a time, occurred twice daily, and ceased after a month entirely. In the month of June he complained for the first and only time of a vertex headache. After this he enjoyed good health until August 31, when I first saw him.

On the previous day he had pushed a heavy barrow for some distance uphill, and almost immediately after his right arm commenced to twitch exactly in the same way as his face had done formerly. The twitchings lasted for a few minutes, and if he happened to hold anything in his right hand at the time he unconsciously let it fall. His grasp was good and equal to the left; there was no loss of power, but marked anæsthesia was occasionally present up to the elbow during and for some time after the spasms. Knee-jerk slightly plus on right side. The heart sounds were normal, urine also; also the blood-vessels. No optic changes in the fundus. There was no specific history. The attacks always followed any active exertion, and never occurred in bed or during rest.

He was told to desist from hard work and put on iodide mixture with a blue pill twice a week. From this time till January 26, 1908, he enjoyed the best of health. He had never vomited. On January 26 he quite suddenly lost the power in his right hand, with paralysis of the whole of the lower right half of the face, but he could speak freely. Three days after the right leg became paralysed, and he could say yes or no, but nothing else. Two days after he lost these words, but was quite conscious of what was going on, had the newspapers read to him, and took an intelligent interest in his surroundings. There was very slight blurring of the right optic disc at this time.

By a process of exclusion intra-cranial tumour was diagnosed. He lingered till February 11, when he died after being unconscious for a week. There was, therefore, a history of almost a year, with quite a sudden collapse and a typical hemiplegia. The post-mortem examination, for which I am indebted to Professor Sutherland, University College, Dundee, was exceptionally interesting, and is quoted in full. It is as follows:—

The brain as received shows some fulness in the left hemisphere, the tissue in the central parts conveying the impression of increased density on palpation. After hardening in Kaiserling, a series of frontal (coronal) sections is made. A tumour is revealed in the substance of the left hemisphere having an antero-posterior measurement of two inches, a horizontal measurement varying from one to two inches and a vertical measurement varying from one to one and a half inches. The central part of the growth is revealed in a section taken half an inch behind the level of the optic chiasma (that is three inches from the anterior limit of the hemisphere). It is situated above and to the left of the lateral ventricle, involving mainly the white substance, but extending downwards by the side of the ventricular wall for about half an inch, invading the upper part of the caudate nucleus. There is also an extension horizontally to the left, the growth reaching to within quarter of an inch of the cortex. The tumour-tissue is firm in its peripheral part; soft, homogeneous, necrotic or hæmorrhagic in the central portion. Anteriorly the tumour tends to approach the vertex. Its anterior limit, corresponding to the anterior extremity of the lateral ventricle, is markedly hæmorrhagic. In its backward extension the tumour-tissue is firmer and less readily differentiated from the cerebral substance.

The growth throughout bounds the upper and left side of the left lateral ventricle, an origin from the wall of which is probable. The growth microscopically has the structure of a very cellular glioma.

